# FABP5, a Novel Immune-Related mRNA Prognostic Marker and a Target of Immunotherapy for Multiple Myeloma

**DOI:** 10.3389/fmed.2021.667525

**Published:** 2021-06-24

**Authors:** Haipeng Jia, Xiaofen Zhang, Xinxin Liu, Ruifang Qiao, Yan Liu, Sulong Lv, Hongbo Zhu, Jie Wang, Qiuhong Kong, Hong Zhang, Zhirong Zhang

**Affiliations:** ^1^Department of Hematology, The Second Affiliated Hospital of Shandong First Medical University, Taian, China; ^2^Respiratory Intensive Care Unit, Taian City Central Hospital, Taian, China

**Keywords:** multiple myeloma, FABP5, immunotherapy, prognosis, therapeutic target, immune microenvironment

## Abstract

**Objective:** Multiple myeloma is an incurable hematological malignancy. It is imperative to identify immune markers for early diagnosis and therapy. Here, this study analyzed immune-related mRNAs and assessed their prognostic value and therapeutic potential.

**Methods:** Abnormally expressed immune-related mRNAs were screened between multiple myeloma and normal bone marrow specimens in the GSE47552 and GSE6477 datasets. Their biological functions were then explored. Survival analysis was presented for assessing prognosis-related mRNAs. CIBERSORT was utilized for identifying 22 immune cell compositions of each bone marrow specimen. Correlation between FABP5 mRNA and immune cells was then analyzed in multiple myeloma.

**Results:** Thirty-one immune-related mRNAs were abnormally expressed in multiple myeloma, which were primarily enriched in B cells-related biological processes and pathways. Following validation, FABP5 mRNA was a key risk factor of multiple myeloma. Patients with its up-regulation usually experienced unfavorable outcomes. There were distinct differences in the infiltration levels of B cells naïve, B cells memory, plasma cells, T cells CD4 naïve, resting memory CD4 T cells, activated memory CD4 T cells, Tregs, resting NK cells, M0 macrophages, M1 macrophages, M2 macrophages, and neutrophils between multiple myeloma and normal samples. FABP5 mRNA had correlations to B cells memory, B cells naïve, dendritic cells activated, macrophages M0, macrophages M1, macrophages M2, neutrophils, activated NK cells, resting memory CD4 T cells, CD8 T cells and Tregs.

**Conclusion:** Collectively, our data showed that FABP5 mRNA was related to immune microenvironment, which could be a target of immunotherapy and prognostic marker for multiple myeloma.

## Introduction

Multiple myeloma is a heterogenous and incurable neoplasm of plasma cells, accounting for 1% of malignancies and around 10% of all hematological malignancies (1). Stem cell transplantation, proteasome inhibitors and immunosuppressive agents are the main therapeutic strategies, which have prolonged survival time of multiple myeloma subjects (2). Multiple myeloma is a multi-step disease, initially characterized by asymptomatic monoclonal gammopathy (MGUS). MGUS accounts for 1% of the adult population, and ~1% of MGUS patients turn into malignant multiple myeloma every year (3). Despite the remarkable clinical outcomes, nearly all subjects experience recurrence. Hence, it still requires novel therapeutic approaches.

The survival, growth as well as proliferation of myeloma cells depend upon the bone marrow microenvironment (4, 5). The microenvironment contains a variety of cell types, including hematopoietic cells (B cells, T cells, natural killer cells, bone marrow-derived suppressor cells, and osteoclasts) and non-hematopoietic cells (marrow stromal cells, osteoblasts, and endothelial cells) (6). These cells secrete various factors, thereby promoting the migrative as well as proliferative behaviors of multiple myeloma cells (7–9). Multiple myeloma progress closely associates with abnormal innate and adaptive immune systems (10). There is evidence that the residual immune cell dysfunction in the tumor microenvironment can lead to the suppression of the host's anti-tumor immune function (11–13). In the normal microenvironment, effector cells can produce a powerful anti-tumor response. Nevertheless, tumor cells often protect themselves from the host immune system by inhibiting the anti-tumor immunity of their surrounding microenvironment. The sophisticated interplays between immune microenvironment and this disease may affect the clinical responses to immuno-oncology therapies such as thalidomide, lenalidomide (14) as well as pomalidomide (15). Early studies have mainly established the role of the bone marrow microenvironment in the pathological process of multiple myeloma, but the immune components of the microenvironment have not received enough attention. The immune response is a dynamic and complex process. It is of importance for understanding the main factors that contribute to the immunosuppressive environment in multiple myeloma (16). Herein, we focused on abnormally expressed immune-related mRNAs in multiple myeloma. Among them, high expression of FABP5 mRNA displayed poor outcomes of subjects. FABP5 is a member of the FABP family, which is overexpressed in several cancers such as prostate cancer (17). Increasing evidence has demonstrated that FABP5 is involved in tumor progression (18). Nevertheless, the biological roles of FABP5 remain indistinct in multiple myeloma. Our further analysis demonstrated that FABP5 dysregulation was in relationship with immune microenvironment of multiple myeloma, indicating that FABP5 was an underlying immunotherapeutic target for multiple myeloma.

## Materials and Methods

### Data Preparation

From the Gene Expression Omnibus (GEO) database (https://www.ncbi.nlm.nih.gov/gds/), microarray data of bone marrow specimens from 5 normal and 41 multiple myeloma subjects were retrieved from the GSE47552 dataset on the GPL6244 platform. Furthermore, microarray expression profiling of 15 normal and 103 multiple myeloma bone marrow samples was obtained from the GSE6477 dataset on the GPL96 platform. The gene expression profiles from the GSE47552 and GSE6477 datasets were utilized for differential expression analysis. Probe IDs were transformed to gene symbols, followed by log2 transformation. The GSE4452 dataset contained complete follow-up information of 65 multiple myeloma subjects. Moreover, the GSE4204 dataset comprised survival information of 538 multiple myeloma patients. The GSE4452 and GSE4204 datasets were used for survival analysis.

### Immune-related mRNAs

Two thousand four hundred and ninety-eight immune-related mRNAs were obtained from the Immunology Database and Analysis Portal (ImmPort) database (https://www.immport.org/home) (19). Supplementary Table 1 listed the list of immune-related mRNAs.

### Differential Expression Analyses

The differences in immune-related mRNAs between multiple myeloma and normal bone marrow specimens from the GSE47552 and GSE6477 datasets were analyzed through “limma” package (20). These mRNAs with false discovery rate (FDR) <0.05 and |log2fold change| > 1 displayed differential expression. Abnormally expressed immune-related mRNAs were intersected between the GSE47552 and GSE6477 datasets.

### Functional Annotation Analyses

Gene Ontology (GO) as well as Kyoto Encyclopedia of Genes and Genomes (KEGG) functional annotation analyses for abnormally expressed immune-related mRNAs were carried out *via* “clusterProfiler” package (21). GO terms contained biological process (BP), molecular function (MF), and cellular component (CC). Terms with FDR <0.05 were significantly enriched. The enrichment results of the top 10 terms were depicted by “ggplot2” package.

### Survival Analysis

Univariate cox regression analyses were presented for assessment of which mRNAs displayed significant relationships to outcomes of multiple myeloma in the GSE4204 dataset through “survival” package. Hazard ratio (HR), 95% confidence interval (CI) as well as *p*-value were separately determined. The mRNAs with *p* <0.05 were survival-related mRNAs. The mRNAs with HR > 1 were protective factors, while those with HR <1 were risk factors. Then, patients in the GSE4204 dataset were separated into high and low expression subgroups in line with the median value of each survival-related mRNA. Kaplan-Meier curves between two subgroups were conducted. Log-rank test was utilized for evaluating whether there were differences in overall survival between subgroups. The prognostic implications of survival-related mRNAs were verified in the GSE4452 dataset.

### Cell-Type Identification by Estimating Relative Subsets of RNA Transcripts (CIBERSORT)

Twenty-two immune cell compositions of bone marrow specimens were characterized based on the mRNA expression profiling from the GSE4204 dataset through the CIBERSORT algorithm (http://cibersort.stanford.edu/) (22). The perm was set at 1000. Specimens with *p* < 0.05 were retained for the analyses. These immune cells contained B cells naïve, B cells memory, plasma cells, CD8 T cells, T cells CD4, naïve T cells, CD4 memory resting, T cells CD4 memory activated, T cells follicular helper, T cells regulatory (Tregs), T cells gamma delta, NK cells resting, activated NK cells, monocytes, macrophages M0, macrophages M1, macrophages M2, dendritic cells resting, dendritic cells activated, mast cells resting, mast cells activated, eosinophils, and neutrophils. Correlation analyses were carried out between different immune cell compositions in each sample. The differences in fractions of 22 immune cells between normal and multiple myeloma bone marrow specimens were assessed through Mann-Whitney *U*-test. Correlations between FABP5 expression and immune cell fractions were determined among bone marrow samples by Pearson correlation analysis.

### Statistical Analyses

R language (https://www.r-project.org/) and corresponding packages were carried out for statistical analyses.

## Results

### Abnormal Expression of Immune-Related mRNAs in Multiple Myeloma

**Figure 1:**
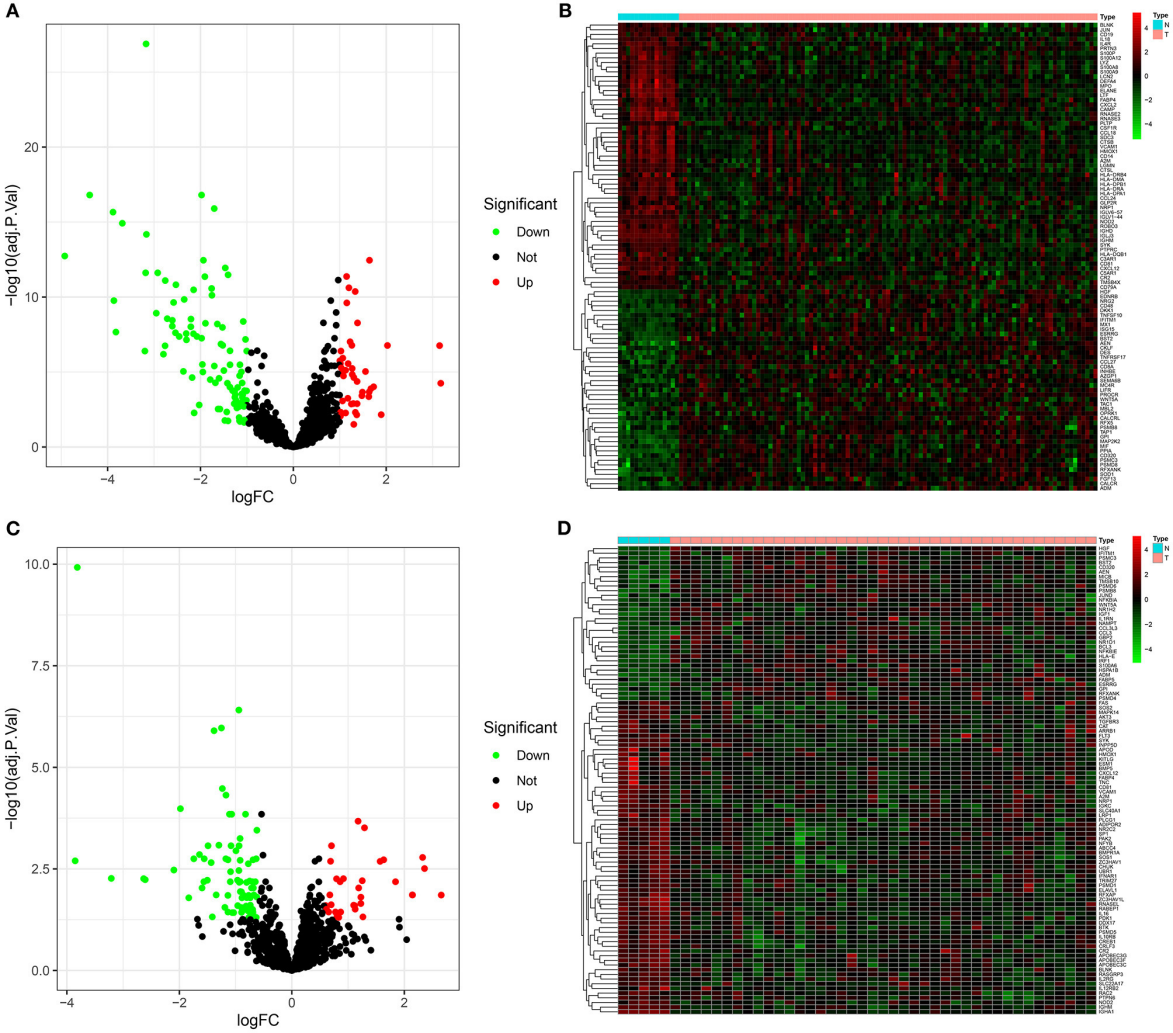
Abnormally expressed immune-related mRNAs between multiple myeloma and normal bone marrow specimens. **(A)** Volcano plots for up-regulated (red) and down-regulated (green) immune-related mRNAs in multiple myeloma than normal specimens in the GSE6477 dataset. **(B)** Hierarchical clustering analyses for differential expression patterns of immune-related mRNAs in multiple myeloma and normal samples in the GSE6477 dataset. N indicates normal samples and T indicates multiple myeloma samples. **(C)** Volcano plots for highly (red) and lowly (green) expressed immune-related mRNAs in multiple myeloma than normal specimens in the GSE47552 dataset. **(D)** Heat map for abnormal expression patterns of immune-related mRNAs in multiple myeloma and normal samples in the GSE47552 dataset.

In the GSE6477 dataset, 139 immune-related mRNAs displayed abnormal expression between multiple myeloma and normal bone marrow specimens (Supplementary Table 2). Among them, 96 mRNAs showed down-regulation and 43 mRNAs exhibited up-regulation in multiple myeloma than normal bone marrow specimens (Figure 1A). The expression patterns of these mRNAs possessed conspicuous differences between multiple myeloma and normal subgroups (Figure 1B). Table 1 listed the top 10 up- and down-regulated mRNAs in multiple myeloma. In the GSE47552 dataset, there were 33 up-regulated and 81 down-regulated immune-related mRNAs (Figure 1C). Supplementary Table 3 listed the detailed information of 114 immune-related mRNAs. These mRNAs were distinguished multiple myeloma from normal bone marrow specimens (Figure 1D). Table 2 displayed the top 10 up- and down-regulated mRNAs for multiple myeloma. Thus, immune-related mRNAs may participate in multiple myeloma progress.

**Table 1:** The top 10 up- and down-regulated mRNAs for multiple myeloma in the GSE6477 dataset.

**ID**	**logFC**	**Average Expression**	***t***	***P*-value**	**FDR**	**B**
EDNRB	3.17564	9.427753	4.73802	5.98E-06	5.62E-05	3.309497
DKK1	3.150724	9.880272	6.177481	9.22E-09	1.73E-07	9.570701
CD320	2.027545	8.59685	6.184919	8.90E-09	1.70E-07	9.605457
AZGP1	1.892662	7.210442	3.212207	0.001691	0.006999	−2.021902
INHBE	1.729437	6.682186	4.588078	1.11E-05	9.68E-05	2.718796
HGF	1.670569	8.019395	4.501167	1.58E-05	0.00013	2.382487
AEN	1.64025	9.420039	9.046557	3.19E-15	3.53E-13	24.13246
ISG15	1.633916	10.83147	4.363766	2.72E-05	0.000213	1.860149
ADM	1.625863	10.82119	4.152458	6.19E-05	0.00044	1.079927
ESRRG	1.554311	7.725752	5.205616	8.10E-07	9.27E-06	5.232271
RNASE3	−3.16441	7.972736	−9.838842	4.17E-17	6.60E-15	28.39332
RNASE2	−3.171877	8.870617	−15.60233	1.19E-30	1.32E-27	59.00869
CD14	−3.18157	6.364479	−8.654979	2.66E-14	2.43E-12	22.0487
HLA-DRA	−3.197716	8.593233	−5.971892	2.45E-08	4.00E-07	8.618598
LTF	−3.683454	6.394645	−10.17587	6.52E-18	1.20E-15	30.21743
LYZ	−3.821531	7.269164	−6.663122	8.59E-10	2.17E-08	11.88284
IGHM	−3.865049	11.58396	−7.703542	4.21E-12	1.73E-10	17.08209
ELANE	−3.88425	5.808598	−10.51921	9.80E-19	2.17E-16	32.07939
IGLJ3	−4.386589	10.19574	−11.08725	4.26E-20	1.57E-17	35.16217
IGHD	−4.923749	10.40866	−9.207839	1.32E-15	1.83E-13	24.99565

**Table 2:** The top 10 up- and down-regulated mRNAs for multiple myeloma in the GSE47552 dataset.

**ID**	**logFC**	**Average Expression**	***t***	***P*-value**	**FDR**	**B**
CCL3	2.656638	8.795874	3.56051	0.000872	0.013934	−0.89655
IFITM1	2.359537	9.723628	4.272571	9.57E-05	0.003099	1.171609
ADM	2.325244	8.837015	4.62326	3.06E-05	0.001651	2.249141
HGF	2.145919	6.485812	3.566572	0.000856	0.013856	−0.879762
CCL3L3	2.141869	9.028911	3.576213	0.000832	0.013819	−0.853034
GBP2	1.843693	5.860666	3.907079	0.000304	0.006551	0.087163
IGF1	1.633367	7.666151	4.504304	4.52E-05	0.001888	1.880063
IRF1	1.569844	9.045278	4.42257	5.90E-05	0.002064	1.628528
PSMB8	1.291786	7.809183	5.30784	3.09E-06	0.000307	4.428956
WNT5A	1.263791	5.512224	2.879846	0.006015	0.048083	−2.669174
BMPR1A	−1.642355	5.335115	−4.698554	2.39E-05	0.001405	2.48447
PDK1	−1.743202	9.479215	−4.553513	3.85E-05	0.001781	2.032324
SLC40A1	−1.832581	7.009903	−3.460474	0.001172	0.016285	−1.171216
CD81	−1.981184	7.328404	−5.804343	5.63E-07	0.000104	6.050917
RASGRP3	−2.097604	7.852103	−4.238532	0.000107	0.003372	1.068818
VCAM1	−2.607187	6.369076	−4.016176	0.000216	0.005826	0.40622
CXCL12	−2.634773	7.51967	−4.051305	0.000193	0.0055	0.509833
IGKC	−3.210252	9.68074	−4.067062	0.000184	0.005416	0.556443
IGHA1	−3.814438	7.833927	−10.45918	9.24E-14	1.20E-10	20.88334
IGHM	−3.852736	9.121807	−4.457749	5.26E-05	0.002004	1.736577

### Abnormal Expression of Immune-Related mRNAs Is Involved in B Cell-Related Pathways

**Figure 2:**
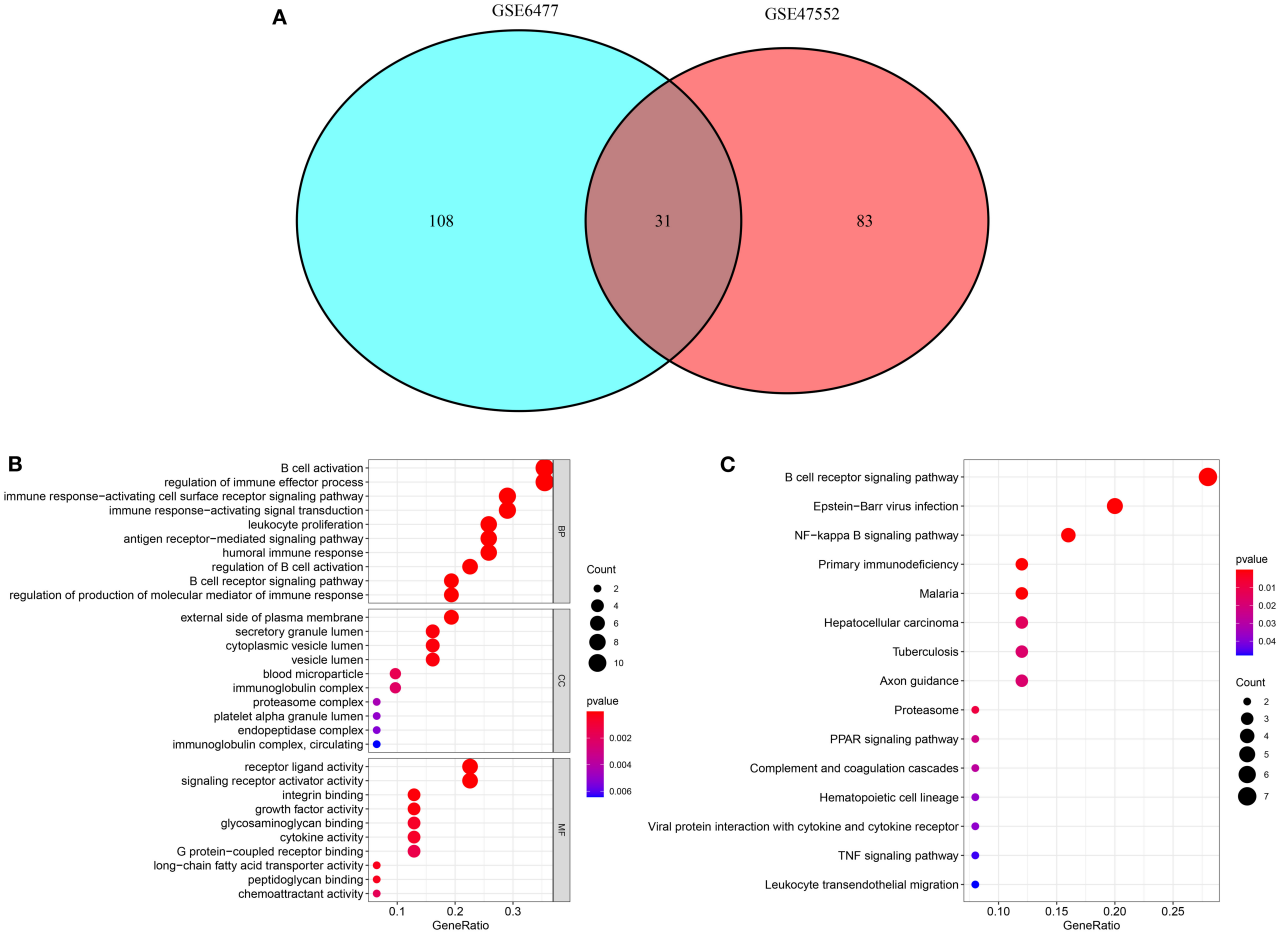
Functional annotation analyses for dysregulated immune-related mRNAs. **(A)** Venn diagram for common dysregulated immune-related mRNAs in the GSE6477 and GSE47552 datasets. **(B)** The top 10 GO results enriched by common mRNAs, composed of biological process (BP), cellular component (CC), and molecular function (MF) terms. **(C)** KEGG pathways involved in common mRNAs. The size of the bubble is proportional to the count of enriched genes. The more the color tends to red, the smaller the *p*-value.

Following intersection of abnormally expressed immune-related mRNAs in the GSE6477 and GSE47552 datasets, 31 common mRNAs were obtained, including CD81, PSMB8, CR2, ABCC4, ADM, IGHM, SYK, IFITM1, RASGRP3, IGKC, CXCL12, VCAM1, ESRRG, AEN, SLC22A17, NRP1, A2M, NOD2, BLNK, CD320, HGF, CCL8, BST2, RFXANK, HMOX1, PSMC3, FABP5, GPI, FABP4, WNT5A, and CD19 (Figure 2A). The biological functions that they were involved in were analyzed in depth. These mRNAs primarily participated in B cell-related biological processes such as B cell activation, regulation of immune effector process, immune response-activating cell surface receptor signaling pathway, immune response-activating signal transduction, leukocyte proliferation, antigen receptor-mediated signaling pathway, humoral immune response, regulation of B cell activation, B cell receptor signaling pathway, and regulation of production of molecular mediator of immune response (Figure 2B). They were mainly involved in regulating the cellular components of external side of plasma membrane, secretory granule lumen, cytoplasmic vesicle lumen, vesicle lumen, blood microparticle, immunoglobulin complex, proteasome complex, platelet alpha granule lumen, endopeptidase complex, and immunoglobulin complex, circulating and the like. Moreover, they possessed the molecular functions of receptor ligand activity, signaling receptor activator activity, integrin binding, growth factor activity, glycosaminoglycan binding, cytokine activity, G protein-coupled receptor binding, long-chain fatty acid transporter activity, peptidoglycan binding, and chemoattractant activity and the like. The pathways enriched by these common mRNAs were analyzed. In Figure 2C, they principally participated in B cell receptor signaling pathway, Epstein-Barr virus infection, NF-kappa B signaling pathway, primary immunodeficiency, malaria, hepatocellular carcinoma, tuberculosis, axon guidance, proteasome, PPAR signaling pathway, complement and coagulation cascades, hematopoietic cell lineage, viral protein interaction with cytokine and cytokine receptor, TNF signaling pathway and leukocyte transendothelial migration.

### FABP5 mRNA as an Immune-Related Prognostic Marker of Multiple Myeloma

**Figure 3:**
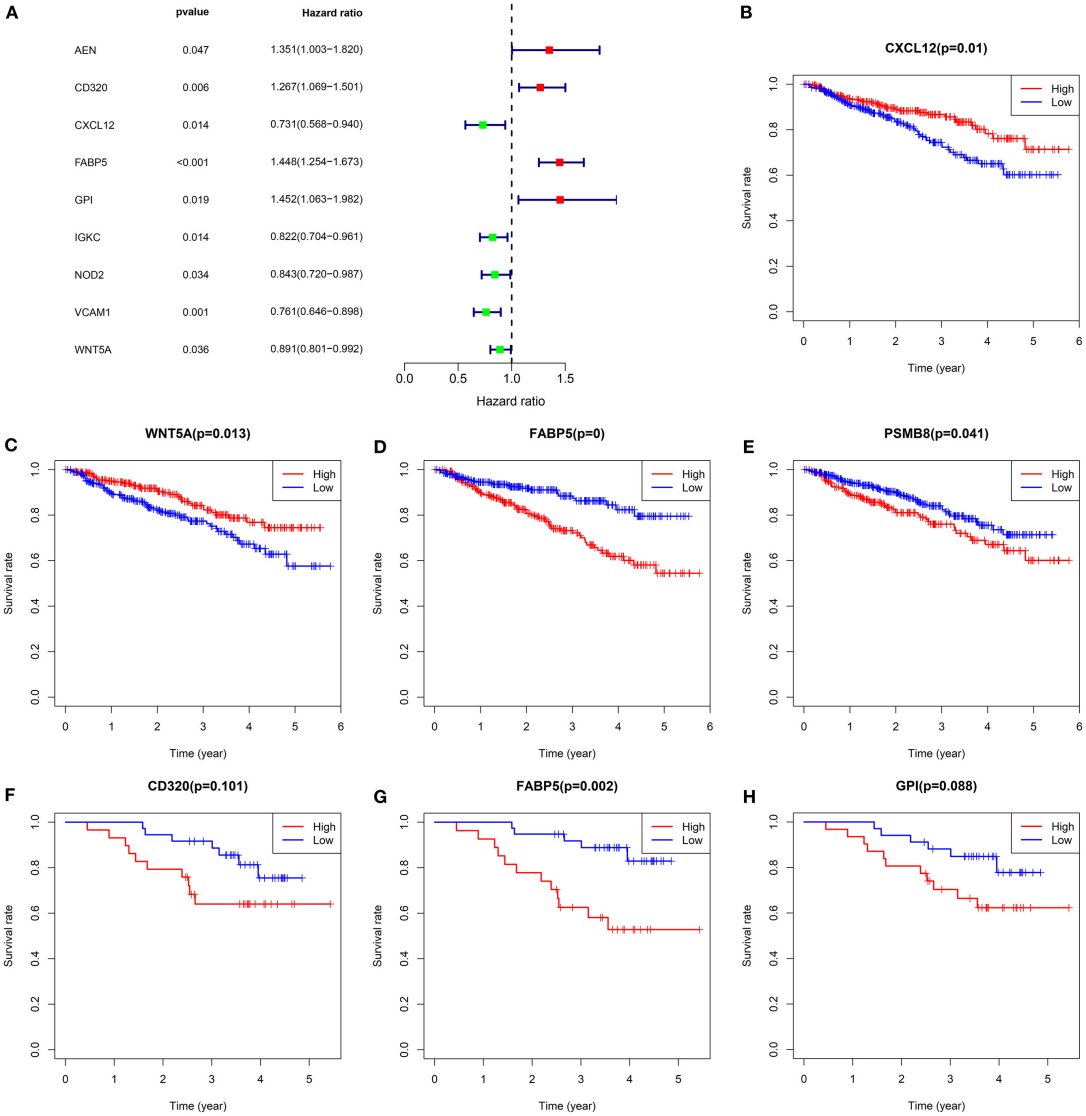
Survival analysis for common dysregulated immune-related mRNAs in multiple myeloma. **(A)** Forest plots for univariate cox regression analysis of common dysregulated immune-related mRNAs in the GSE4204 dataset. Kaplan-Meier survival curves of **(B)** CXCL12, **(C)** WNT5A, **(D)** FABP5, and **(E)** PSMB8 expression among multiple myeloma subjects in the GSE4204 dataset. Kaplan-Meier survival curves of **(F)** CD320, **(G)** FABP5, and **(H)** GPI expression among subjects in the GSE4452 dataset. *P*-values for log-rank test. Red indicates high expression group while blue indicates low expression group.

As depicted by univariate cox regression analyses, among 31 common dysregulated immune-related mRNAs, AEN (HR: 1.35132218004432, 95% CI: 1.00336950593063–1.81993933788734, *p*-value: 0.0474670415899411), CD320 (HR: 1.2666572113788, 95% CI: 1.06872217896594–1.50125123508741, *p*-value: 0.0063988822159732), FABP5 (HR: 1.44824784649006, 95% CI: 1.25386429924306–1.67276620454803, *p*-value: 4.74125786831674e-07), and GPI (HR: 1.45171072863824, 95% CI: 1.06338050769041–1.98185317899107, *p*-value: 0.0189309122327046) were risk factors of multiple myeloma subjects in the GSE4204 dataset (Figure 3A). Meanwhile, CXCL12 (HR: 0.730888940033441, 95% CI: 0.568482755798023–0.939691903078592, *p*-value: 0.0144802428352221), IGKC (HR: 0.82233697739277, 95% CI: 0.704030330155922–0.960524107303318, *p*-value: 0.0135803713395964), NOD2 (HR: 0.842773450385212, 95% CI: 0.719652065331376–0.986959008235654, *p*-value: 0.0337646407355474), VCAM1 (HR: 0.761232879031875, 95% CI: 0.6455778262167–0.897607496086219, *p*-value: 0.00117564947725508) and WNT5A (HR: 0.891231234461453, 95% CI: 0.800514277560811–0.992228540507633, *p*-value: 0.035517571353358) were protective factors of multiple myeloma. In the GSE4204 dataset, our Kaplan-Meier survival analyses demonstrated that low expression of CXCL12 (*p*-value: 0.009800177; Figure 3B) and WNT5A (*p*-value: 0.012647237; Figure 3C) displayed more unfavorable outcomes for multiple myeloma patients. Meanwhile, subjects with high FABP5 (*p*-value: 5.22E-05; Figure 3D) and PSMB8 (*p*-value: 0.040988621; Figure 3E) expression often experienced shorter survival time. In the GSE4452 dataset, we found that subjects with high CD320 (*p*-value: 0.100794819; Figure 3F), FABP5 (*p*-value: 0.002496168; Figure 3G) and GPI (*p*-value: 0.087747724; Figure 3H) were indicative of poorer outcomes compared to those with their low expression. Combining above data, FABP5 mRNA was a key immune-related prognostic marker for multiple myeloma.

### Tumor Immune Cells in Multiple Myeloma Bone Marrow Specimens

**Figure 4:**
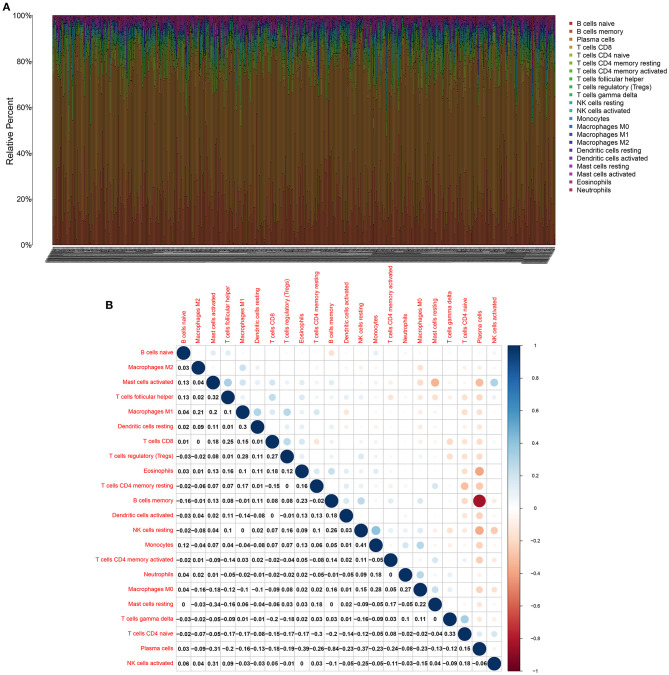
CIBERSORT identifies tumor immune cells in multiple myeloma bone marrow specimens. **(A)** Stacked bar chart for the proportions of 22 kinds of immune cells in each multiple myeloma bone marrow samples. Each type of immune cell is identified by a unique color. **(B)** The correlation between the proportions of immune cells in multiple myeloma bone marrow specimens. Blue indicates negative correlation and red indicates positive correlation. The larger the correlation coefficient, the bigger the bubble.

CIBERSORT was employed to identify the mixture of B cells naïve, B cells memory, plasma cells, CD8 T cells, T cells CD4, naïve T cells, CD4 memory resting, T cells CD4 memory activated, T cells follicular helper, Tregs, T cells gamma delta, NK cells resting, activated NK cells, monocytes, macrophages M0, macrophages M1, macrophages M2, dendritic cells resting, dendritic cells activated, mast cells resting, mast cells activated, eosinophils and neutrophils in multiple myeloma bone marrow specimens (Figure 4A). Correlations between the proportions of 22 immune cells in each multiple myeloma bone marrow sample were further analyzed. In Figure 4B, plasma cells displayed a strongly negative correlation to B cells memory (*r* = −0.84). Monocytes possessed a positive relationship with NK cells resting (*r* = 0.41).

### Abnormal Expression of Immune Cells in Multiple Myeloma Bone Marrow

**Figure 5:**
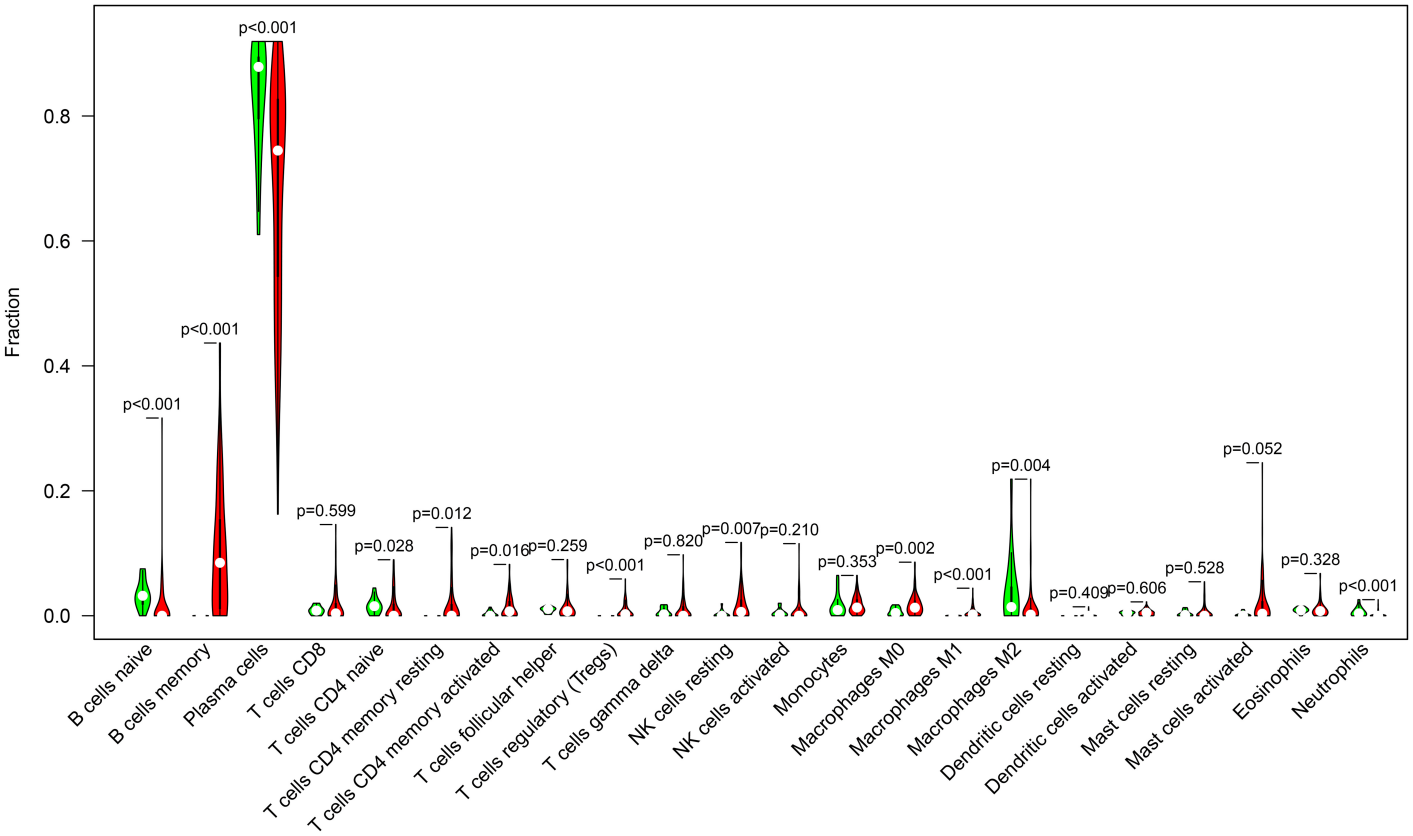
Violin diagram for abnormal expression of immune cells in multiple myeloma (red) and normal (green) bone marrow specimens in the GSE6477 dataset.

We compared the infiltrations of immune cells in multiple myeloma and normal bone marrow specimens in the GSE6477 dataset. Data showed that there were significant differences in the infiltration levels of B cells naïve (*p* < 0.001), B cells memory (*p* < 0.001), plasma cells (*p* < 0.001), T cells CD4 naïve (*p* = 0.028), resting memory CD4 T cells (*p* = 0.012), activated memory CD4 T cells (*p* = 0.016), Tregs (*p* < 0.001), resting NK cells (*p* = 0.007), M0 macrophages (*p* = 0.002), M1 macrophages (*p* < 0.001), M2 macrophages (*p* = 0.004), and neutrophils (*p* < 0.001) between multiple myeloma and normal bone marrow specimens (Figure 5).

### FABP5 mRNA Exhibits Correlations to Immune Microenvironment of Multiple Myeloma

**Figure 6:**
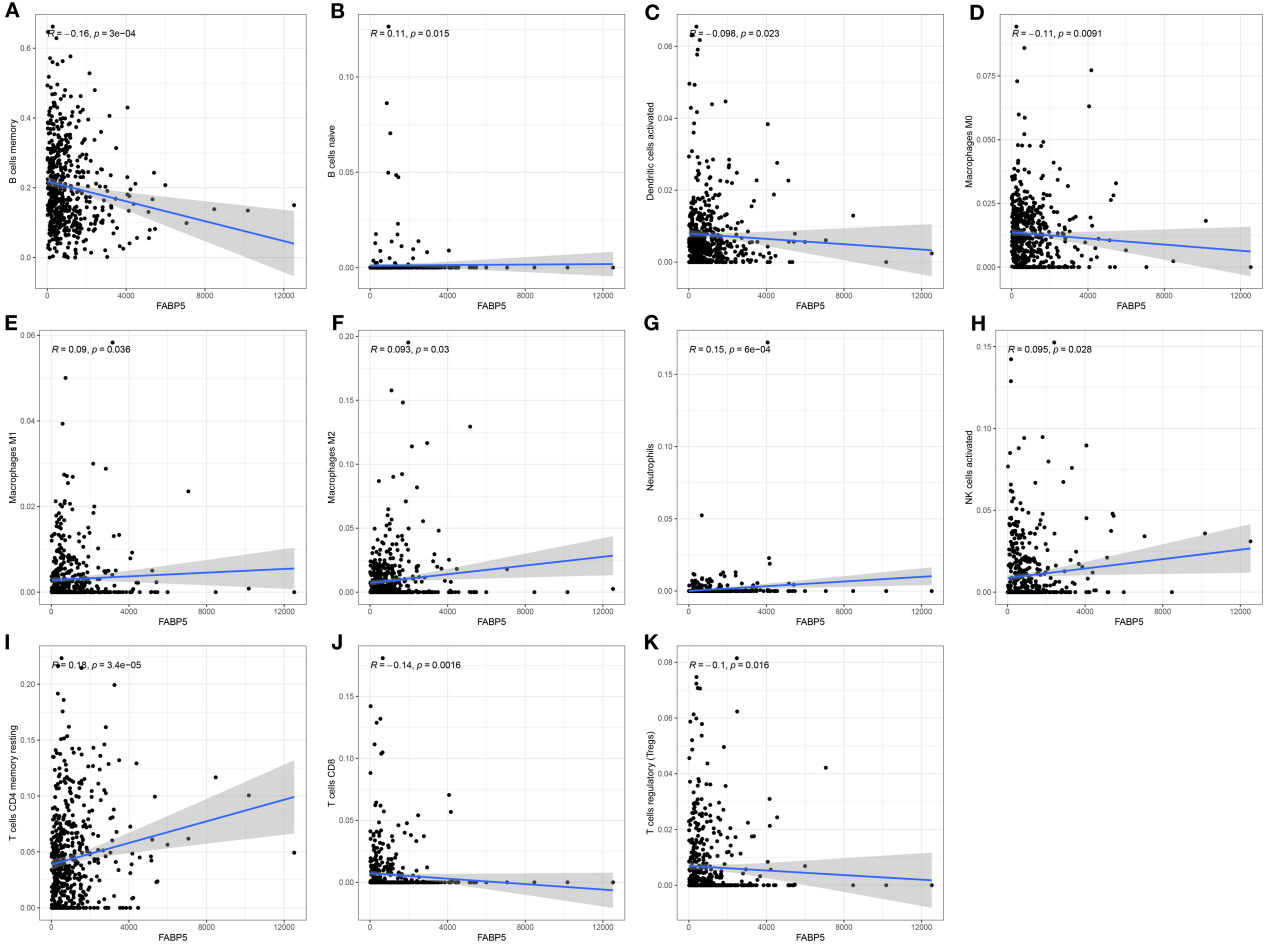
Associations between FABP5 mRNA and infiltrations of immune cells in multiple myeloma bone marrow specimens. **(A)** B cells memory; **(B)** B cells naïve; **(C)** dendritic cells activated; **(D)** macrophages M0; **(E)** macrophages M1; **(F)** macrophages M2; **(G)** neutrophils; **(H)** activated NK cells; **(I)** resting memory CD4 T cells; **(J)** CD8 T cells; **(K)** Tregs.

**Figure 7:**
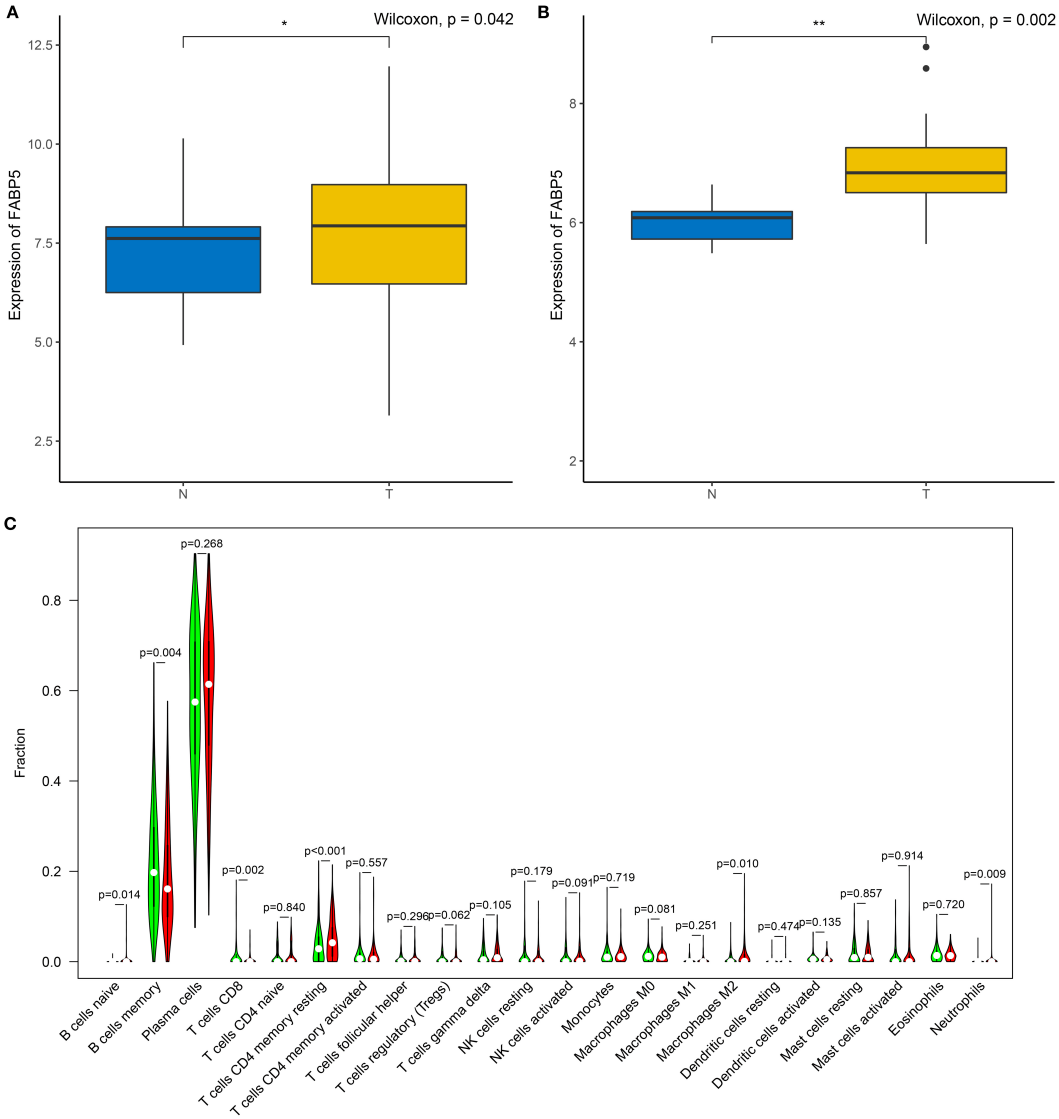
Association between FABP5 mRNA and immune microenvironment of multiple myeloma. **(A,B)** Expression of FABP5 mRNA in multiple myeloma and normal bone marrow specimens in the **(A)** GSE6477 and **(B)** GSE47552 datasets. **(C)** Infiltration levels of immune cells between high and low FABP5 expression groups. Red: high FABP5 expression group; green: low expression group. *P < 0.05; **P < 0.01.

The relationships between FABP5 mRNA expression and infiltrations of immune cells were assessed in multiple myeloma bone marrow specimens. In Figure 6A, FABP5 mRNA displayed a negative association with infiltration of B cells memory (*r* = −0.16, *p* = 3e-04). A positive correlation between FABP5 mRNA and B cells naïve was detected in multiple myeloma bone marrow specimens (*r* = 0.11, *p* = 0.015; Figure 6B). FABP5 mRNA was negatively correlated to levels of dendritic cells activated (*r* = −0.098, *p* = 0.023; Figure 6C) and macrophages M0 (*r* = −0.11, *p* = 0.0091; Figure 6D). Furthermore, FABP5 mRNA exhibited positive correlations to infiltrations of macrophages M1 (*r* = 0.09, *p* = 0.036; Figure 6E), macrophages M2 (*r* = 0.093, *p* = 0.03; Figure 6F), neutrophils (*r* = 0.15, *p* = 6e-04; Figure 6G), activated NK cells (*r* = 0.095, *p* = 0.028; Figure 6H) and resting memory CD4 T cells (*r* = 0.18, *p* = 3.4e-05; Figure 6I). We also found that there were negative associations between FABP5 mRNA and infiltrations of CD8 T cells (*r* = −0.14, *p* = 0.0016; Figure 6J) and Tregs (*r* = −0.1, *p* = 0.016; Figure 6K). Both in the GSE6477 (Figure 7A) and GSE47552 (Figure 7B) datasets, FABP5 mRNA was up-regulated in multiple myeloma compared to normal bone marrow specimens (*p* = 0.042 and 0.002). There were higher levels of B cells naïve (*p* = 0.014), T cells CD4 memory resting (*p* < 0.001), macrophages M2 (*p* = 0.010) and neutrophils (*p* = 0.009) as well as lower levels of B cells memory (*p* = 0.004) and T cells CD8 (*p* = 0.002) in the high FABP5 expression group compared to its low expression group (Figure 7C).

## Discussion

Multiple myeloma is an aggressive and incurable hematological malignancy, manifested by the malignant proliferation of abnormal plasma cells (23). Despite the advancement of treatment strategies, more therapeutic targets are still required for multiple myeloma. Here, we identified FABP5 as a novel immune-related mRNA as well as a prognostic marker for this malignancy.

Through integrative analyses of the GSE6477 and GSE47552 datasets, this study screened 31 common dysregulated immune-related mRNAs in multiple myeloma. These mRNAs were primarily enriched in B cell-related biological functions such as B cell activation, B cell receptor signaling pathway, plasma membrane. These data were indicative that they participated in the pathogenesis of multiple myeloma. Different patients' outcomes vary greatly. Thus, the establishment of a precise prognostic evaluation system to identify patients with different risks, especially for high-risk patients, is essential for clinicians to formulate overall treatment strategies, and can provide an important reference for patients and their families to understand the disease and achieve better management. Among all dysregulated immune-related mRNAs, AEN, CD320, FABP5, and GPI were risk factors for multiple myeloma, while CXCL12, IGKC, NOD2, VCAM1, and WNT5A were protective factors for multiple myeloma. Among them, CXCL12 up-regulation could be induced by HIF-2α in multiple myeloma plasma cells, thereby reducing migration as well as adhesion to mesenchymal stromal cells (24). CXCL12 is a target for reversing cellular adhesion-induced drug resistance against multiple myeloma (25). NOD2/CARD15 variant is correlated to the sensitivity of multiple myeloma bone marrow cells to bortezomib (26). Sialyltransferase inhibitor may suppress relationships between E-selectin, VCAM1, and MADCAM1, thereby prolonging survival time of multiple myeloma patients (27). WNT5A is an abundant growth factor upon myeloma bone marrow specimens (28). After multiple dataset verification, FABP5 was a key prognostic factor for multiple myeloma. Subjects with FABP5 up-regulation were indicative of more unfavorable outcomes. Hence, it was a risk factor of this disease. Nevertheless, the malignant roles of FABP5 mRNA remain undefined in multiple myeloma. Liu et al. reported that FABP5 was a stage-related prognostic factor of this malignancy (29). Its roles have been expounded in other malignancies. For instance, FABP5 is a critical driving factor of metastatic prostate carcinoma (30). Furthermore, it may promote lymph node metastases for cervical carcinoma *via* reprogramming fatty acid metabolisms (17). Up-regulated FABP5 induced by fatty acid actuates hepatocellular carcinoma progress (31). FABP5 may elevate PI3K/AKT-mediated proliferation of renal carcinoma cells (18). Combining previous research, FABP5 mRNA might be a potential immunotherapeutic target of multiple myeloma. However, it remains unclear about which immunotherapy (dendritic cell vaccine, CAR T cells, or CAR NK cells) is good for targeting FABP5. Furthermore, whether FAPB5 targeted treatment causes any toxicity in patients with multiple myeloma requires further analysis. Thus, in our future studies, we will validate the therapeutic effects of FABP5 mRNA therapy in multiple myeloma by experiments.

There was a strongly negative correlation of plasma cells and B cells memory in multiple myeloma bone marrow specimens, indicating that there was interplay between plasma cells and B cells memory. There were distinct differences in the infiltration levels of B cells naïve, B cells memory, plasma cells, T cells CD4 naïve, resting memory CD4 T cells, activated memory CD4 T cells, Tregs, resting NK cells, M0 macrophages, M1 macrophages, M2 macrophages, and neutrophils between multiple myeloma and normal samples. Macrophages are innate immune cells that play a role in the host's own defense and maintenance of tissue homeostasis. Macrophages maintain the growth of myeloma cells through cell-to-cell contact-dependent behaviors and non-contact-mediated mechanisms in the bone marrow compartment, while enhancing the protective effect of mesenchymal stem cells on tumor cells (32). As an important part of the bone marrow microenvironment, through the connection between macrophages and tumor cell activation signal pathways, they can inhibit the protease pathway and block drug-induced apoptosis (33). Similar to solid tumors, multiple myeloma cells regulate immune molecules in the bone marrow microenvironment to adapt them to the growth of myeloma itself (34). The main immunosuppressive mechanisms during tumor progression include regulation of the expansion of immune cells, dysfunction of antigen presenting cells and suppression of immune effector cells (like effector T cells, NK cells) (35). Our data were indicative that FABP5 mRNA displayed correlations to B cells memory, B cells naïve, dendritic cells activated, macrophages M0, macrophages M1, macrophages M2, neutrophils, activated NK cells, resting memory CD4 T cells, CD8 T cells and Tregs, suggesting the relationships of FABP5 mRNA with immune microenvironment of multiple myeloma.

However, the prognostic value of FABP5 mRNA will be confirmed in a multicenter multiple myeloma cohort. Moreover, more experiments should be verified the functions of FABP5 in multiple myeloma progress and immune microenvironment.

## Conclusion

This study screened 31 abnormally expressed immune-related mRNAs for multiple myeloma. These mRNAs were primarily involved in B cell-related pathways. After verification in multiple datasets, patients with FABP5 mRNA usually unfavorable outcomes. There was crosstalk between immune cells in multiple myeloma bone marrow specimens. FABP5 mRNA displayed significant correlations to infiltrations of immune cells. Taken together, FABP5 mRNA could be a promising therapeutic target as well as prognostic marker in multiple myeloma.

## Data Availability Statement

The datasets presented in this study can be found in online repositories. The names of the repository/repositories and accession number(s) can be found in the article/Supplementary Materials.

## Author Contributions

ZZ conceived and designed the study. HJ, XZ, XL, RQ, and YL conducted most of the experiments, data analysis, and wrote the manuscript. SL, HZhu, JW, QK, and HZha participated in collecting data and helped to draft the manuscript. All authors reviewed and approved the manuscript.

## Conflict of Interest

The authors declare that the research was conducted in the absence of any commercial or financial relationships that could be construed as a potential conflict of interest.

## Supplementary Material

Supplementary Table 1 A list of 2,498 immune-related mRNAs.

Supplementary Table 2 One hundred and thirty-nine abnormally expressed immune-related mRNAs for multiple myeloma in the GSE6477 dataset.

Supplementary Table 3 One hundred and thirty-nine abnormally expressed immune-related mRNAs for multiple myeloma in the GSE47552 dataset.

## Abbreviations

GEO: Gene Expression Omnibus
ImmPort: Immunology Database and Analysis Portal
GO: Gene Ontology
KEGG: Kyoto Encyclopedia of Genes and Genomes
BP: biological process
MF: molecular function
CC: cellular component
HR: hazard ratio
CI: confidence interval
CIBERSORT: Cell-type Identification By Estimating Relative Subsets of RNA Transcripts
Tregs: T cells regulatory.
